# Commentary: From Old World monkeys to New World humans—Evolved protection from tick bites and bioprosthetic material

**DOI:** 10.1016/j.xjon.2021.03.019

**Published:** 2021-04-01

**Authors:** Antonia Schulz, Edward Buratto, Igor E. Konstantinov

**Affiliations:** a Department of Cardiac Surgery, Royal Children's Hospital, Melbourne, Australia; b Department of Paediatrics, University of Melbourne, Melbourne, Australia; c Heart Research Group, Murdoch Children's Research Institute, Melbourne, Australia; d Melbourne Centre for Cardiovascular Genomics and Regenerative Medicine, Melbourne, Australia

**Central Message**

Next-generation bioprosthetic valves for transcatheter and surgical implantation should have optimal tissue biocompatibility to minimize inflammatory immune response.

**Figure:**
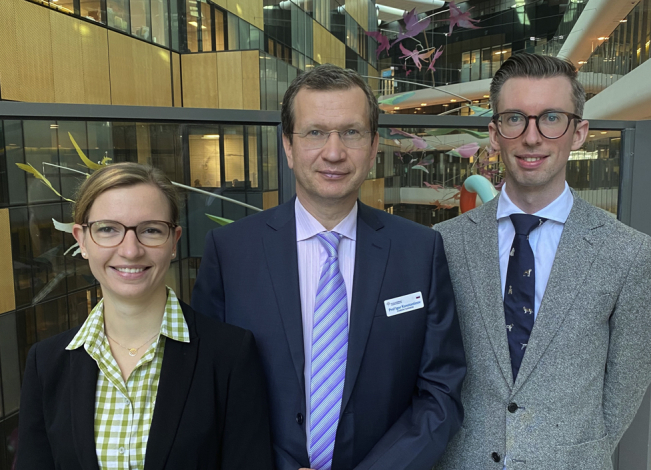
Drs Antonia Schulz, Igor E. Konstantinov, and Edward Buratto

In a fascinating twist of evolution, Old World monkeys, apes, and humans developed a remarkable inactivation of the alpha-1,3-galactosyltransferase gene, which, in turn resulted in a unique recognition of alpha-gal epitope so that high titers of antibodies against this antigen are produced. Although in nature this evolutionary advantage protected us against tick bites and other arthropod vector-borne diseases, in clinical practice, it may cause a spectrum of immune response from immediate anaphylaxis to xenotransplantation to delayed calcifications of bioprosthetic material. This phenomenon, including delayed anaphylaxis to red meat consumption, is known as alpha-gal syndrome.^1^^,^^2^

See Article page 85.

In the last decade, an increasing variety of patients have benefitted from transcatheter aortic valve implantation (TAVI) as an alternative to surgical aortic valve replacement.^3^^,^^4^ In this issue of the *Journal*, Veraar and colleagues^5^ challenge us to improve the biocompatibility of bioprosthetic heart valves used for TAVI, as it might be a limiting factor for durability. The study demonstrates significantly increased serum concentrations of alpha-gal–specific antibodies, augmented complement activity, and nonspecific inflammation in 27 patients 3 months after TAVI compared with patients undergoing a MitraClip procedure, who served as controls. Similar xenograft-specific immune response has been observed after surgical bioprosthetic valve replacement.^6^^,^^7^ Alpha-gal epitopes were also identified in decellularized bioprosthetic material when complete decellularization was not achieved.^8^ The resulting humoral response leads to activation of the complement system, triggering endothelial cell dysfunction, platelet aggregation, and promotes calcification.^2^^,^^9^, ^10^, ^11^

Although the presented study of Veraar and colleagues^5^ did not explore any relationship between the degree of immunogenic response and valve durability, other groups were able to show an association of anti–alpha-gal antibodies and premature bioprosthetic valve degeneration.^12^ Furthermore, several experimental studies demonstrated a connection between anti–alpha-gal antibodies and the calcification process in valvular bioprosthesis.^10^^,^^11^ Less immunogenic materials and improved processing methods have already been described to increase biocompatibility and to prevent an immunologic response to the xenogenic valve tissue.^9^^,^^11^ It appears that a proper understanding of alpha-gal syndrome is important to improve the longevity of bioprosthetic material^13^, ^14^, ^15^, ^16^, ^17^ in patients with a wide range of congenital and acquired heart disease.
